# Systematic Understanding of Recent Developments in Bacterial Cellulose Biosynthesis at Genetic, Bioprocess and Product Levels

**DOI:** 10.3390/ijms22137192

**Published:** 2021-07-03

**Authors:** Gizem Buldum, Athanasios Mantalaris

**Affiliations:** 1 Department of Life Sciences, Imperial College London, London SW7 2AZ, UK; g.buldum11@imperial.ac.uk; 2 Wallace H. Coulter Department of Biomedical Engineering, Georgia Institute of Technology, Atlanta, GA 30332, USA

**Keywords:** bacterial cellulose, synthetic biology, bioprocessing, synthetic circuit modeling

## Abstract

Engineering biological processes has become a standard approach to produce various commercially valuable chemicals, therapeutics, and biomaterials. Among these products, bacterial cellulose represents major advances to biomedical and healthcare applications. In comparison to properties of plant cellulose, bacterial cellulose (BC) shows distinctive characteristics such as a high purity, high water retention, and biocompatibility. However, low product yield and extensive cultivation times have been the main challenges in the large-scale production of BC. For decades, studies focused on optimization of cellulose production through modification of culturing strategies and conditions. With an increasing demand for BC, researchers are now exploring to improve BC production and functionality at different categories: genetic, bioprocess, and product levels as well as model driven approaches targeting each of these categories. This comprehensive review discusses the progress in BC platforms categorizing the most recent advancements under different research focuses and provides systematic understanding of the progress in BC biosynthesis. The aim of this review is to present the potential of ‘modern genetic engineering tools’ and ‘model-driven approaches’ on improving the yield of BC, altering the properties, and adding new functionality. We also provide insights for the future perspectives and potential approaches to promote BC use in biomedical applications.

## 1. Bacterial Cellulose: What We Know So Far

Bacterial cellulose (BC) is one of the distinctive materials produced by nature. Its ultrapure and nanofibrillar structure differentiates itself from plant cellulose. BC is well known for being strong and flexible with high water holding capacity reaching up to ~90% of its weight. Therefore, it comes as no surprise that BC attracts significant attention and numerous approaches have been pursued for research and development of BC.

In the last few decades, bacteria capable of BC synthesis and the characterization of BC have been well-documented. Many members of *Acetobacteraceae*, especially those in *Komagataeibacter* genus, over-produce bacterial cellulose extracellularly, in the form of pellicle at the liquid–air interface in liquid culture [1]. BC is not crucial for survival but possesses a survival advantage by aiding in attachment, adherence, and subsequent colonization of a substrate. Most bacteria produce extracellular polysaccharides, which form an envelope-like structure around cells. Similarly, cellulose-producing bacteria are embedded in the cellulose network, which supports the population at the liquid–air interface. The cellulose layer helps nutrient supply for embedded bacteria, as their concentration in the polymer matrix is significantly enhanced due to highly adsorptive structure. Moreover, cellulose layer protects cellulose-producing cells against critical changes such as pH, water content, and accumulation of toxic substances. It has been reported that the cellulose layer protects bacteria from ultraviolet radiation [2,3].

BC is often characterized by its high purity. It is naturally produced free from the other substances such as pectin and lignin that are co-produced by plant cells. The purification process for plant cellulose has mechanical and chemical separation steps including logging, debarking, chipping, mechanical pulping, screening, chemical pulping, and bleaching, which require high energy and the whole purification process itself is environmentally unfriendly [4]. On the other hand, BC obtained after fermentation contains only some impurities such as cells and/or the medium components. Therefore, the purification process is extremely simple compared to that of plant cellulose. Widely used purification processes of BC include the treatment with alkali (sodium hydroxide or potassium hydroxide), organic acids like acetic acid, or repeated washing of the mixtures with the reverse osmosis water [5].

The biocompatibility of BC nanofibers when combined with its high water holding capacity makes BC suitable for wound dressings and artificial skin production. BC allows the transfer of medicine into the wound while serving as an efficient physical barrier against external infection. BC has been also used for numerous biomedical and tissue-engineering applications, as well as production of high-quality papers, diaphragms for audio speakers, and polymer composites [6,7,8,9,10,11].

The unique properties of BC arise from its structure. Although both bacterial and plant cellulose have an identical molecular formula, BC differs from plant cellulose in terms of microfibrillar structure. BC is composed of glucose units connected through β-1,4 glycosidic bonds. These molecules are covalently linked through acetal functions between the equatorial -OH group of C4 and the C1 carbon atom. As a result, cellulose is a linear-chain polymer with a large number of hydroxyl groups. The polar -OH groups form many hydrogen bonds with oxygen atoms on the same or on a neighbour chain. These hydrogen bonds between and within cellulose chains constitute stable crystalline regions and give the structure more stability and strength. Two forms of cellulose are produced by *Komagataeibacter*: (i) cellulose I, the ribbon-like polymer, and (ii) cellulose II, the thermodynamically more stable amorphous polymer [12]. Two allomorphs of cellulose (cellulose I and cellulose II) of BC are significantly different in their stability, crystallinity, and H-bonding patterns. Cellulose I is less stable and more crystalline due to the highly ordered H-bonding patterns of its parallel glucan chains.

The metabolic pathway of cellulose biosynthesis by *Komagataeibacter* has been well-documented [2,13,14]. It is a multi-step reaction involving individual enzymes, catalytic complexes, and regulatory proteins. If glucose is used as a carbon source, the biosynthesis pathway constitutes of four key enzymatic steps: (i) phosphorylation of glucose by glucokinase, (ii) isomerization of glucose-6-phosphate (Glc-6-P) to glucose-1-phosphate (Glc-1-P) by phosphoglucomutase, (iii) synthesis of UDP-glucose (UDPGlc) by UDPG-pyrophosphorylase (UGPase), and (iv) cellulose synthase reaction. UDPGlc is a common molecule in many organisms, which is the direct cellulose precursor, however; not many of these organisms are cellulose producers. UGPase is approximately 100 times more active in cellulose producers than that of non-cellulose producing bacteria, hence it is thought to play an important role in cellulose synthesis [15].

Cyclic diguanylic acid (c-di-GMP) has also an important role in the synthesis of BC as an allosteric activator for the cellulose synthase. BcsA is a membrane protein with a glycosyltransferase domain and PilZ domain. c-di-GMP binds to the PilZ domain of the BcsA subunit of *G. xylinus* cellulose synthase. Studies showed that the biosynthesis of cellulose in *G. xylinus* and other cellulose-synthesizing bacteria was actually promoted by the increased level of c-di-GMP [16,17].

In addition to enzymatic steps, structural assembly of cellulose fibers has two intermediary steps. The first stage of BC biosynthesis represents the polymerization of UDP-Glucose units by the formation of β-1,4-glucan chains in the inner membrane. This stage is followed by the secretion of the polymer and assembly of the fibrils. Fibrils are formed between the outer and cytoplasm membranes of the cell. The β-1,4-glucan chains are spun through cellulose export components to form protofibrils, which are approximately 2–4 nm in diameter. A ribbon shaped microfibril of approximately 80 nm is assembled from these protofibrils and then crystallization and assembly of the fibrils [18].

The core machinery of cellulose biosynthesis is catalyzed by cellulose synthase (bcs) operon, which is flanked by accessory genes (*cmcAx*, *ccpAx*, and *bglAx*) [19]. BcsA is an integral inner membrane protein with transmembrane (TM) domains [20]. BcsB is a periplasmic protein attached to BcsA and contains carbohydrate binding domains that chaperone the synthesized glucan chain through the periplasm [21]. Cellulose nanofibers are synthesized by the joint action of BcsA (the catalytic subunit) and BcsB using UDP-glucose as a substrate. The fibers are then secreted through outer membrane pores formed by BcsC, which shows similarity to the proteins involved in membrane channels or pore formation [22]. BcsD appears to be a non-essential gene for BC biosynthesis; however, cellulose production is reduced by 90% without it [23]. BcsD seems to assist in the proper orientation of the linear terminal complexes along the longitudinal axis of the cell, indicating the BcsD participates in the final level of the hierarchical assembly of cellulose.

Numerous ancillary genes are involved in the regulation, synthesis, crystallization, and export of BC [2,22,24]. CcpAx, Cmcax, and BlgAx proteins flanking bcs operon are not essential for the BC biosynthesis, but they are involved in the correct glucan chains formation. *ccpAx* encodes a cellulose-complementing protein, *cmcax* and *bglxA* encode endo-β-1,4-glucanase (EC 3.2.1.4) and β-glucosidase (EC 3.2.1.21), respectively, both of which assist cellulose biosynthesis by hydrolyzing tangled glucan chains when a failure in chain arrangement occurs. It has been hypothesized that CcpAx is involved in the structural organization of the terminal complexes, cooperating with BcsD. Additionally, Römling and Galperin proposed a model for the organization of the entire BC synthase complex based on crystal structure data of the *Rhodobacter sphaeroides* BcsA-BcsB complex, the BcsC-like AlgK-AlgE protein complex of *Pseudomonas aeruginosa*, and the BcsD protein of *K. xylinus* [22].

## 2. Recent Advances

**Figure 1:**
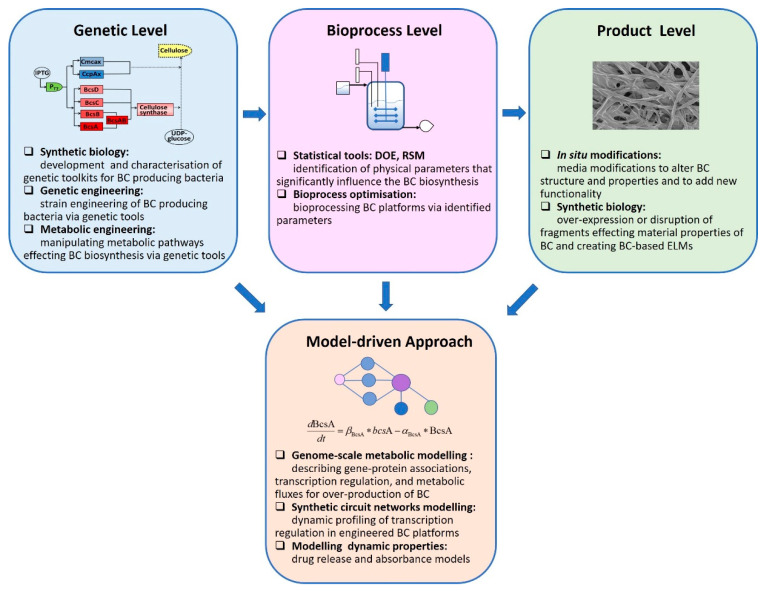
Summary of recent research focusing on BC biosynthesis platforms.

For many decades, the studies mostly concentrated on the optimization of cellulose production through modification of culturing strategies and conditions. Different medium components and additives (ethanol, vitamins, agar, sodium alginate), culturing conditions (pH, temperature, dissolved oxygen), and fermentation techniques (static, agitated, bioreactors) have been studied [3,4]. More recently, intensive studies have focused on strain engineering, manipulating the cellulose biosynthesis pathway, and improving BC functionality. Researchers are exploring new strategies via synthetic biology, metabolic engineering, bioprocess engineering, and model-driven approaches. In this review, we discuss and present the recent progress on BC biosynthesis categorizing at different levels: genetic level, bioprocess level, and product level as well as modeling approaches targeting each level (Figure 1).

### 2.1. Genetic Level

#### 2.1.1. Genetic Engineering and Synthetic Biology Approaches

Microbial production of chemicals depended on only nature’s ability for many years. Thanks to the rapid development of genetic engineering, it became possible to systematically engineer different living organisms to produce various commercially valuable chemicals such as biofuels, biomaterials, therapeutics, or food and beverage ingredients. In the early years, research has focused largely on well-studied hosts, including *Escherichia coli* and *Saccharomyces cerevisiae*, due to the availability of wide information on their genetics as well as the availability of a wide range of toolkits [25,26].

When it comes to cellulose production, genetic modifications targeting *Komagataeibacter* spp. were initially limited to a few vector backbones (pSA, pBBR122) and focusing on addition or deletion of genes to improve cellulose production [27,28] (Table 1). One of the first studies were focusing on developing strains that are capable of carbon assimilation from sucrose [28,29,30] or lactose [31], to explore more cost-effective carbon sources than glucose (Table 1). In order to improve the O_2_ utilization of the cells, *Vitreoscilla* hemoglobin (VHb) expression was driven by the constitutive *bla* promoter on a vector derived from pBBR122 in the cellulose-producing *K. xylinus*. The expressed VHb enhanced cell growth (50% higher than that of wild type), leading to an increased cellulose production after 6 days of culturing (6 g/L for wild type, 11 g/L for VHb(+) mutant) [32]. The same group constructed a recombinant *A. xylinum* expressing d-Amino acid oxidase (DAAO) of *Rhodosporidium toruloides*. The cells successfully produced DAAO but also self-immobilized by cellulose nanofibers; however, the activity of DAAO was around 10% [33]. Another important study was redesigning the flow of cellular metabolites to incorporate N-acetylglucosamine (GlcNAc) sugar residues into glucan chains during the biosynthesis [34]. An operon composed of three genes from *Candida albicans* (AGM1, NAG5, and UAP1) responsible for UDP-GlcNAc synthesis was expressed under the control of *bla* promoter in *K. xylinus* again derived from pBBR122. The modified strain was able to convert the monomer of chitin, N-acetylglucosamine (GlcNAc), into activated cytoplasmic UDPGlcNAc. When both glucose and GlcNAc supplemented, UDP-glucose and UDPGlcNAc were accessible to cellulose synthase to synthesize a cellulose-chitin copolymer. The copolymer contained over 18-fold more GlcNAc, as compared to the control BC. It was displayed improved degradability *in vivo* as chitin is susceptible to degradation by animal lysozymes. However, partially or fully substituting GlcNAc for glucose significantly lowered total cellulose production in both engineered and wild-type cells. With glucose-fed cultures, modified BC production (1.8 ± 0.2 mg/mL culture medium) was 35% lower than wild-type BC production.

Due to slow growth leading to extensive cultivation times (ranging from 3 days to 8 days), *Komagataeibacter* genus was not presenting a great candidate for time and cost-efficient production of cellulose. Scientists suggested engineering well-studied hosts could have high potential for exploring new cellulose-producing stains to overcome these limitations. *E. coli* represents a strong candidate for accomplishing BC production in new platforms due to its rapid growth kinetics [35]. Initially, reconstitution of cellulose synthase in *E. coli* was achieved by heterogeneous co-expression of BcsA, BcsB, and DGC (diguanyl cyclase). Despite the successful reconstitution of cellulose synthase, amorphous cellulose was obtained in a non-native cellulose II structure, indicating the importance of the genes responsible for export and crystallization (BcsC and BcsD) [36].

Buldum et al. achieved cellulose production in *E. coli* by heterogeneous expression of complete bcsABCD operon along with the upstream operon (CcpAx and Cmcax) [37]. The product represented a remarkable fiber structure with a diameter ranging from 10 to 20 μm. Cellulose fibers were notable in the culture as early as 3 h after IPTG induction and the total culture time was 18 h, leading to a time and energy efficient production process. One notable challenge of this process was the metabolic stress on *E. coli* caused by expression of membrane associated bcs proteins. Commonly used *E. coli* BL21 was not able to tackle this task even at low culturing temperatures and inducer concentrations due to inactive accumulation of bcs proteins, whereas derivative C41 was able to accomplish BC production. The volumetric productivities were in the same range with that of *Komagataeibacter* due to the slow growth of *E. coli* at low culturing temperature (22 °C), which is necessary for functional expression of bcs and upstream operon.

More recently, engineering of *Komagataeibacter* for endogenous and heterologous expression has become easier due to the progress in synthetic biology toolkits introduced into the field. Modular DNA programming by using these toolkits made it possible to add new functionalities and to produce BC with modified material properties. In 2016, Florea et al. isolated a strain of *K. rhaeticus* from Kombucha tea (*K. rhaeticus* iGEM) that can grow in low-nitrogen conditions [38]. The authors developed a synthetic biology toolkit that allows transformation, controlled expression of constitutive and inducible transgenes, and control over endogenous gene expression of this strain. They tried many plasmid systems and found out a total of 5 plasmids showing replication: pSEVA321 and 331 and 351 pBAV1K-T5-sfGFP, and pBla-Vhb-122. The authors studied the expression of seven reporter proteins and characterized constitutive and inducible promoters. In 2019, Teh et al. presented an expanded genetic toolkit for synthetic biology applications in *Acetobacteraceae*. Authors characterized the performance of multiple natural and synthetic promoters (11 constitutive promoters from Anderson family, two inducible promoters: P_Lux_ and P_Bad_), ribosome binding sites (36 novel RBS mutants), terminators (five natural intrinsic terminators and five synthetic terminators), and degradation tags (five variants of the natural *E. coli* ssrA tag) in three different strains, (*Gluconacetobacter xylinus* ATCC 700178, *Gluconacetobacter hansenii* ATCC 53582, and *K. rhaeticus* iGEM). In addition to that, the authors implemented CRISPR interference (CRISPRi) for the first time in *G. hansenii* ATCC 53582, targeting endogenous acs operon (acsAB and acsD). A significant decrease in *acsAB* expression (by more than two-fold) was detected. For the *acsAB*-targeting sgRNA, the yield decreased by around 15%, while for the *acsD*-targeting sgRNA, the yield dropped by around 5%. They further applied their tools to synthesize a biodegradable cellulose-chitin copolymer mimicking the system previously established by Yadav et al. The authors showed that generating modified cellulose with variable chitin content is possible by tuning the rate of GlcNAc incorporation via high or low expression plasmids. Their data revealed strain specific and common design rules for the precise control of gene expression in these industrially relevant bacterial species [39].

In another study, CRISPRi was used to downregulate *galU* in *K. xylinus* CGMCC 2955 (Table 1) [40]. The authors first explored the metabolic regulatory mechanism of cellulose structure under different oxygen tensions and analyzed the transcriptome data of *K. xylinus* under varying oxygen tensions (GenBank: CP024644.1). They found that oxygen-enriched conditions downregulated the expression level of genes (*hk*, *pgm*, and *galU*) involved in BC biosynthesis, which suggested that this may be the reason for the significant decrease in BC production. *galU* regulates the production of UGPase, the activity of which in cellulose-producing strain is 100 times higher than in nonproducing strain. The expression of *galU* in the strains with sgRNA-dCas9 complex targeted to the sites of − 34, 329, 615, and 771 from the starting codon, decreased by 96.80%, 67.51%, 62.68%, and 30.25%, respectively. The macromolecular structure of cellulose was also affected by tuned *galU* expression, which is discussed in Section 2.3.

#### 2.1.2. Metabolic Engineering and Synthetic Biology Approaches

Metabolic engineering and synthetic biology depend on each other to progress rather than work separately. Synthetic biology aims to establish libraries (promoters, coding sequences, terminators, transcriptional factors), the assembly strategies, genetic circuits, obtain quantitative information for model development that can predict the behaviour of biological systems. Metabolic engineering applies this information for the optimization of cellular processes and the manipulation metabolic fluxes, to produce a compound of interest, preferably cheap and simple [48].

Extensive studies on the cellulose biosynthesis pathway revealed that glucose cannot be utilized only for cellulose synthesis because of the amounts of byproducts produced in bypasses, which limits the yield of cellulose [49]. This suggested metabolic pathway modulation to reduce the related byproducts such as gluconic acid can be an important approach to improve bacterial cellulose production [50].

Oxidation of glucose into gluconic acid results in lowering pH and rapid loss of glucose from medium in *Komagataeibacter* cultures. Glucose dehydrogenase (GDH), a membrane-bound protein, controls the oxidation of glucose to gluconic acid that decreases the conversion of glucose to bacterial cellulose. To overcome this difficulty, a mutant of *K. xylinus* was generated by knocking-out the membrane bound GDH. Without the membrane bound GDH activity, the mutant GDH-KO strain consumed glucose four times slower than the wild-type. In contrast, the wild-type strain oxidized a large fraction of glucose to gluconic acid that decreased the conversion yield of glucose to BC. The authors showed that the BC production from GDH-KO strain was about 40 and 230% higher than that of wild-type strain in static and shaken culture, respectively [41]. In another similar study, the mutant GD-1 strain was shown to be able to effectively produce 5.0 g/L of BC from a saccharified solution, which was derived from sweet potato pulp by enzymatic saccharification [51].

Another interesting study reported an entire process for development for a sustainable BC nanofiber separator for lithium rechargeable batteries [42]. The authors questioned the absence of *pfkA* gene in *Komagataeibacter species*, indicating that glucose is metabolized through the oxidative pentose phosphate pathway instead of the Embden–Meyerhof–Parnas (EMP) pathway, because of a lack of the *pfkA* gene. Their simulated flux balance analysis with an additional reaction catalyzed by *pfkA* gene showed that *pfkA* is beneficial for both the BC production rate and specific growth rate. Thus a mutant strain harbored the *pfkA* gene fused in chromosome (S.Koma-*pfkA*) was created. Furthermore, they overexpressed CRP under the control of the tac promoter in the S.Koma-*pfkA* strain. CRP is mainly involved in the catabolism of alternate carbon sources and positively regulates the expression of the EMP pathway-related genes (*fba*, *glk*, *pck*, and *pgi*). Compared with Koma, the S.Koma-*pfkA/crp* strain showed increased cellulose production (from 3.5 to 4.5 g/L). Another surprising outcome of this study was the sharp decrease in the yield of gluconic acid (from 64.8 to 39.2%) in S.Koma-*pfkA/crp* cultures compared to that of wild-type. The authors speculated that CRP plays a role not only in the EMP pathway-related genes but also in regulating the multiple gene expression related to byproduct metabolism.

The dissolved oxygen content in the culture medium is critical for cell metabolism and both the yield and quality of BC. As mentioned earlier, the VHb encoding gene was overexpressed in *K. xylinus* to enhance BC production. In another work using the same expression system, the effect of oxygen tension on BC production was investigated in VHb-expressing *K. xylinus* culture (*K*. *xylinus-vgb*^+^). VHb is an oxygen-binding protein widely use to overcome hypoxia for microorganism cultures. *K*. *xylinus* and *K*. *xylinus-vgb*^+^ were statically cultured under hypoxic (10 and 15% oxygen tension in the gaseous phase), atmospheric (21%), and oxygen-enriched conditions (40 and 80%). Under atmospheric conditions, the BC yield of *K*. *xylinus-vgb*^+^ were 22.3% higher than those of *K*. *xylinus*. At oxygen tensions of 10 and 15%, the BC yield of *G*. *xylinus-vgb*^+^ was 26.5 and 58.6% higher than that of *K*. *xylinus*, respectively [43].

### 2.2. Bioprocess Level

When it comes to bioprocessing of BC production, many different medium components, culturing conditions (pH, temperature, dissolved oxygen), and fermentation techniques (static, agitated, bioreactors) have been studied to improve BC productivity. The most efficient approaches are typically supported by the aid of statistical tools for the design of experiments to systematically optimize bioprocess components. Statistical approaches such as response surface methodology (RSM) and design of experiments (DOE) are used to improve the bioprocessing BC systems, yield, and the identification of physical parameters that significantly influence the production. Cerrutti et al. (2015) used the Box–Behnken model and RSM to maximize BC production under static conditions using as parameters concentrations of carbon (grape pomace extract) and nitrogen (corn steep) liquor sources, inoculum size, fermentation time, and temperature. The optimum BC production value predicted by the model (6.7 g/L) and the validation assay result (6.56 g/L), were much higher than the ones in traditional HS medium (1.4 g/L) [52]. Du et al. (used the Plackett–Burman (PB) statistical model to identify physical parameters that influence BC production by *G. xylinus* TJU-S8 isolated from Chinese persimmon vinegar. The result of PB experimental design showed that glucose, initial pH, and ethanol influenced the production of BC significantly. These three parameters were subsequently considered for Box–Behnken analysis followed by the RSM to estimate optimum combination of these parameters for maximizing the production of BC. The optimum combination for the BC yield was found to be the following: 19.142 g/L glucose, 10 g/L yeast extract, 7 g/L peptone, 2.7 g/L Na_2_HPO_4_, 0.5 g/L citric acid, 3.5% ethanol, 30 °C, and initial pH 6.03. The maximum BC yield (4.62 ± 0.17 g/L) in the statistically optimized medium was achieved after 18 h of cultivation, which is 1.46-fold higher than the production in the preliminary non-optimized study (3.20 ± 0.12 g/L) [53].

Abdelraof et al. (2019) used potato peel waste (PPW) hydrolysate as culture media and studied the optimization of five factors: initial pH, medium volume (mL), inoculum size (%), temperature (°C), and incubation time(day) by Taguchi method for the DOE. The results showed that the highest yield of BC was 4.72 g/L, when the initial pH 9.0, temperature 35 °C, medium volume 55 mL, inoculum size 8%, and incubation period 6 days. This result is approximately four times higher than of those observed in the HS medium (1.21 g/L) [54].

In another study, BC production in *G. xylinus* using carob and haricot bean (CHb) medium was statistically optimized by Plackett–Burman design. Eight parameters were evaluated: sugar content of carob (carbon source), protein content of haricot bean (nitrogen source), initial pH, surface area–volume ratio, inoculum ratio, incubation time, citric acid content, and temperature. The most effective parameters for BC production were detected as incubation time, protein amount, and inoculum ratio with contribution of 63.50%#x2013;12.23 and 9.57%, respectively. These significant parameters were optimized by central composite design. Optimal conditions for production of BC in static culture were found as: 2.5 g/L carbon source, 2.75 g/L protein source, 9.3% inoculum ratio, 1.15 g/L citric acid, 2.7 g/L Na_2_HPO_4_, 30 °C incubation temperature, 5.5 initial pH, and 9 days of incubation. This study showed that CHb medium has higher buffering capacity compared to Hestrin and Schramm media [55].

Basu et al. (2019) used a rational approach through Plackett–Burman involving multi-factorial analysis of the following independent parameters viz. (i) carbon source, (ii) carbon concentration, (iii) vessel diameter, and (iv) level-height of the growth medium within the reactor, were considered as the critical process parameters (CPPs) and were used for further process optimization studies. Process parameters helped identify their corresponding interactions and establish a scalable BC bioprocessing platform with yield as high as ~40 g/L for *G. hansenii* 53582 grown on sucrose. The study identified some of the previously overlooked process components. Contrary to the agreement that larger surface area is associated with increased BC production, their data showed insignificant influence of the surface area [56].

### 2.3. Product Level

On the way towards expanding the scale of BC applications, it is crucial to exploit the unique structure and properties of BC to develop novel BC-based nanomaterials with new features. Various functionalization methods for BC-based materials concentrated on the chemical modification or physical coating [57,58]. The macromolecular structure of BC can be controlled by traditional approaches such as the change of cultivation strategy, type of strain, carbon source, and additives [59,60]. However, studies have shown a high potential for manipulating the biosynthesis of BC in order to produce modified BC nanofibers with a controlled composition, morphology, and properties [44,45].

More recently, as an intersection of synthetic biology and materials science, the concept of engineered living materials (ELMs) has evolved [61]. ELMs are engineered materials composed of living biological entities that can form and modulate the material itself [62]. The incorporation of living cells provides many advantages to the materials such as biosensing, self-regenerative, and modulating capabilities [63]. The efforts are also focusing on BC-based ELMs due to their unique properties. In this section, we are going to review in situ biosynthetic approaches and synthetic biology approaches that are explored to open up possibilities for BC with new functionalities.

Gao et al. developed a modification method utilizing the in situ microbial fermentation method combined with 6-carboxyfluorescein-modified glucose (6CF-Glc) as a substrate using *Komagataeibacter sucrofermentans* to produce functional BC with an unnatural characteristic fluorescence. The fluorescence intensity of functional BC was controlled by adjusting the concentration of 6CF-modified glucose (6CF-Glc) in the culture medium. Their approach has potential for modification of other valuable functional materials via this biosynthetic approach [64].

A novel method to glyoxalize BC in situ, during synthesis in the culture medium. The culture medium of *Gluconacetobacter* was modified with a low concentration glyoxal crosslinking agent, thereby allowing effective contact with the BC ribbons. The crystalline structure of cellulose was preserved while a higher hydrophobicity in the BC network was detected. However, the change of the surface from acidic to amphoteric indicates that the reaction between hydroxyl groups of the cellulose and the glyoxal occurred only at the surface [65].

Gelatinized lotus root starch LRS was introduced into the BC culture medium to generate BC/LRS composites. The low viscosity of LRS-containing culture medium allowed unique micro-morphologies with enhanced tensile strength to be generated within BC/LRS composites. The results on in vitro study with cartilage cells showed that the composites presented superior biocompatibility to chondrocytes, which promoted cell viability and accelerated normal cell morphology formation in comparison to BC [66].

Taking one step further, the studies on BC properties, it was demonstrated carboxylmethylcellulose (CMC) was interfering the hydrogen bonding in BC structure, it was hypothesized that CMC should interfere with the production of cellulose nanofibers in different rates according to its degree-of-substitution (DS), either in the crosslinking density or overall network porosity. BC/CMC biocomposites with different DS were prepared in this work by in situ modification of a static culture medium using *Gluconacetobacter*. MTX, a poorly water-soluble drug traditionally used in the treatment of cancer, inflammatory, and autoimmune diseases was incorporated to the BC/CMC membranes in order to optimize the topical treatment of psoriasis, an autoimmune disorder of skin characterized by hyperkeratosis and inflammation. BC/CMC (DS, 0.9) exhibited the lower liquid uptake (up to 11 times lower), suggesting that the more linear structure of the intermediate substitute CMC grade (0.9) was able to interact more strongly with BC, resulting in a denser structure [67].

Synthetic biology approaches are not only employed for engineering the bacteria but also for engineering the material properties of BC. Teh and co-workers discovered that the dominant negative AcsD (dnAcsD) expression causes the production of a dense cellulose matrix as well as thinner fibers than those produced by the wild-type. The authors hypothesized that the presence of dnAcsD might disrupt the crystallization of thicker fibrils after glucan chain extrusion [39].

Jacek et al. investigated the effect of motility genes (*motA* and *motB*) on the structure of cellulose by disruption or overexpression of these genes in *K. hansenii*. Microscopic analysis of the BC membrane produced in overexpressed motility genes exhibited a substantial loosening of intra-membrane structure and fiber thickening was observed [46]. In contrast, disruption of these genes resulted in denser and more compact BC as a result of reduction in motility, and improvement in their mechanical properties [47]. Their study proved that tuning motility-related genes influences the structure of cellulose and results in significantly enlarged pores [46].

To improve another important feature of BC, the water-holding capacity, expression of a biosynthetic gene cluster of colanic acid (wca operon), a water-soluble polysaccharide, was induced in *Enterobacter* sp. FY-07. The results indicated that in situ modified bacterial cellulose hydrogels with different crystallinities, rheological properties, and water-holding capacities were produced by cultivating the engineered strain *Enterobacter* sp. FY-07::tac under different inducer concentrations. The water-holding capacity of the modified bacterial cellulose hydrogel was enhanced slightly by 1.7-fold compared to tBC produced by wild-type [44].

In a previously mentioned study, the expression of *galU* in the strains with sgRNA-dCas9 complex showed that BC membrane porosity increased by 0.5-fold with the repression of *galU* gene. The crystallinity of the BC increased with the rise in the expression level of *galU* up to a certain level. When the expression level of the gene *galU* was 30 times higher than that of the control group, the crystallinity of BC decreased [40].

More recently, BC has drawn significant attention in the field of growing ELMs. Gilbert et al. developed an approach to synthetize functional BC-based ELM using a stable co-culture of *Saccharomyces cerevisiae* and bacterial cellulose-producing *K. rhaeticus* [45]. The authors aimed to engineer *S. cerevisiae* to secrete proteins that form part of grown BC materials to functionalize the materials as yeast has a higher capacity of secreting proteins compared to BC-producing bacteria. They first tested this with the β-lactam hydrolyzing enzyme TEM1 β-lactamase (BLA) and detected clear activity in pellicles co-cultured with BLA-secreting strains even after enzyme-functionalized BC that had been dried and rehydrated. Next, to modify the physical properties of BC, they created a yeast strain with cellulose degradation ability by simultaneous secretion of cellobiohydrolases (CBH1 and CBH2), endoglucanase (EGL2), β-glucosidase (BGL1), and lytic polysaccharide monooxygenases (LPMO). Although the decrease in the BC production was not significant, the properties of BC such as tensile strength and Young’s modulus were weakened. In addition to that, they tested the sense and response ability of BC-based ELM. They combined BC material with a yeast-based biosensor system expressing synthetic transcription factor Z3EV and a green fluorescent protein (GFP) reporter, in which oestrogen steroid hormone β-oestradiol (BED) triggers activation of transcription. BC produced by these co-cultures exhibited a strong GFP signal when exposed to exogenous BED representing that BC-based ELMs can sense and respond to environmental stimuli.

### 2.4. Model-Driven Approaches

Model-driven approaches are progressively becoming crucial for understanding and improving cellular based processes. Systematic investigation of sub-steps and complex interrelationships in a bioprocess leads to a deeper understanding of the entire process. The use of model-driven approaches can minimize the unnecessary experimentation by indicating the most critical components or informative experiments, resulting in a cost and time reduction. Earlier model-driven approaches for understanding BC production systems mainly focused on dynamic bioprocess modeling [68,69]. These studies mostly focused on microbial growth, glucose consumption, and substrate mass transfer prediction. In recent years, the efforts concentrated on metabolic network modeling and synthetic circuit modeling.

#### 2.4.1. Genome-Scale Metabolic Models

Genome-scale metabolic models (GEM) use both the genomic and biochemical information to be particularly useful for systems-level metabolic studies. GEMs computationally describe gene–protein associations, organelle-specific reaction localization, transcriptional/translational regulation, and biomass composition for an entire metabolic network. Reaction stoichiometry and directionality can be simulated to predict metabolic fluxes [70,71]. Zhang et al. (2017) reconstructed the genome-scale metabolic network of *K. nataicola* RZS01 to investigate the distribution of metabolites in the cells cultured in the presence of different carbon sources. Their model employed 771 genes, 2035 metabolites, and 2014 reactions. Constraint-based analysis was used to characterize and evaluate the critical intracellular pathways. The flux balance analysis of central carbon metabolism showed that glycerol led to the highest BC productivity. The minimization of metabolic adjustment algorithm (MOMA) identified eight genes *(pgm*, *ugp*, *cs*, *ct*, *g6pis*, *gadt*, *fadt*, and *udpk*) as potential targets for over-production of BC. In another study, mutant strain of *K. xylinus* was obtained by chemical mutation using DEC (diethyl sulfate) and LiCl. The mutant showed higher BC production and less gluconic acid compared to those of the parent strain. The results revealed a higher flux of TCA cycle in the mutant strain as well as more ATP production from the pentose phosphate pathway (PPP) and TCA cycle [72]. A core metabolic model of *K. hansenii* ATCC 23769, accounts for 68 metabolites and 74 reactions, was developed on genomic and bibliome databases. The model predicted the growing abilities on different substrates and gave insights of the use of minimal medium capable to support BNC production [73].

A comprehensive genome-scale metabolic network of *K. xylinus* was reconstructed based on genome annotation and the data adopted from previously published studies. The network included 640 genes, 783 metabolic reactions, and 865 metabolites. The iMR640 model was used to simulate specific growth rate, glucose consumption, acetic and gluconic acid production, and BC productivity. In the first step, the authors tried to examine the amount of BC production as an objective function by limiting the growth rate and showed 93.17% accuracy. In the second step, growth rate and the production rates of BC and acetic acid were limited, and the model showed 96.76% accuracy [74].

Another genome-scale metabolic model, KxyMBEL1810, was developed to better understand the metabolic features of *K. xylinus* DSM 2325. The KxyMBEL1810 was used to predict essential genes and novel gene over-expression targets for the enhanced CNF production. Random sampling and correlation analysis of the KxyMBEL1810 predicted *pgi* and *gnd* genes as novel overexpression targets for the enhanced BC nanofiber production. The positive effects of individually overexpressing heterologous *pgi* and *gnd* genes on the BC nanofiber were experimentally validated [75].

#### 2.4.2. Synthetic Circuit Modeling

**Figure 2:**
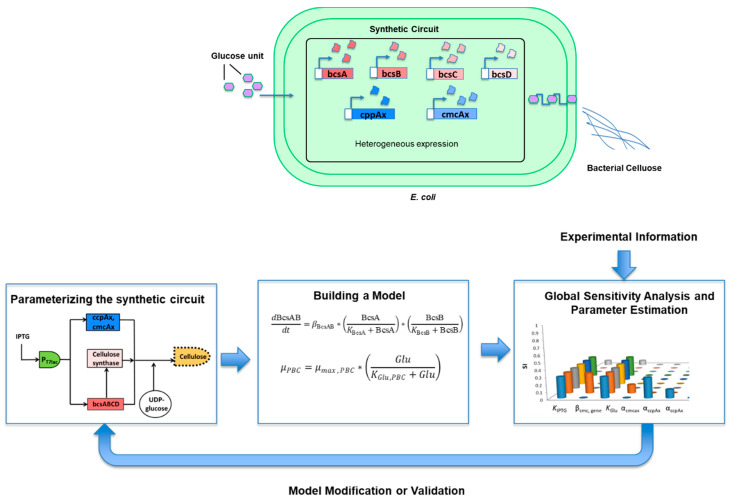
Schematic representation of the synthetic circuit modeling of the BC biosynthetic pathway [77].

Sustainable growth of cell factory development requires not only genome-scale models but also dynamic prediction and scalability of bioprocesses. The typical steady-state assumption entailed ignores dynamic reality, often limiting the applicability of genome-scale models. Successful activation of metabolic networks relies on transcriptional regulation initiating the appropriate metabolic cascades. Prediction of this non-linear dynamic systems behaviour, such as that of engineered gene circuits, has been enabled through rapid advances in model-based designs. Tsipa et al. (2018) developed a systematic bioprocess design tool with high predictive power by linking kinetic gene regulatory network modeling to microbial growth kinetics using consistent gene expression (mRNA) data [76]. This design tool applied to engineered *E. coli* cellulose biosynthesis gene circuit developed by the same research group (Figure 2). The model resulted in dynamic profiling of mRNA gene expression, estimation of enzymes and protein synthesis, and prediction of cellulose biosynthesis. It considers heterogeneous cell populations, caused by the metabolic responses of the host microorganism, and predicts substrate utilization and biomass growth patterns. GSA of the hybrid framework revealed significant parameters associated with gene expression, glucose degradation, and biomass and product formation. Specifically, if the translation efficiencies of Cmcax, CcpAx, BcsC, BcsD, and cellulose synthase were modified, the cellulose biosynthesis would be significantly affected. Furthermore, an increase in plasmid instability would affect glucose degradation and biomass growth patterns and, subsequently, cellulose biosynthesis because of its coupling to microbial growth [77].

#### 2.4.3. Modeling Dynamic Properties

BC has broad use in biomedical applications such as wound dressing, cartilage replacement, artificial skin and blood vessels, tissue regeneration, medical pads, tissue engineering scaffolds, and dental implants. Specifically, for wound dressing applications, it provides the following advantages: high gas exchange, cell respiration permission, good wound exudates absorbing ability, nontoxic, and non-allergenic. Therefore, the research on model-driven approaches also concentrated on drug release and absorbance models of composites materials of BC. Sodium carboxymethylcellulose (NaCMC) and BC composite cross-linked with CA were used as drug delivery matrices containing ibuprofen sodium salt as model drug. The mechanism of ibuprofen sodium salt (IbuNa) release from these composites was investigated using a model proposed by Astarita–Sarti–Cohen–Erneux modified to neglect the volume expansion due to polymer swelling and to consider non-linear diffusion coefficients for drug and solvent. The obtained results suggest that composite films containing only biopolymers (NaCMC and BC) and which are cross-linked with a nontoxic agent (CA) could be used as biopolymeric carriers for drug delivery applications [78].

George et al. (2014) used five different mathematical models to fit the experimental sorption data of hydroxypropyl methyl cellulose (HPMC)-based hybrid nanocomposites reinforced with bacterial cellulose nanocrystals (BCNC). Among these models, the Peleg model was found to be the best fitting model suitable for the composite material [79]. In another study, the released rate of the model drug ampicillin (AP) from the BC-gelatin composite sponges proved to be depended on the initial addition of AP that the diffusional constant (n) determined using the Korsmeyer–Peppas model indicating the AP release from BG composite sponges follows non-Fickian diffusion [80]. The release of the drug from BC is tuned to achieve immediate and controlled delivery by using two drying strategies: freeze-drying and oven-drying. Diclofenac sodium (DCF), a hydrophilic drug, was used as the model drug and was loaded in oven-dried BC (BC-OD-DCF) and freeze-dried BC (BC-FD-DCF) to obtain sustained release and burst release, respectively. The mathematical modeling of drug release kinetics of BC-OD-DCF supports diffusion-driven first-order release from BC-FD-DCF whereas release from BC-OD-DCF shows a supercase II transport, where the buffer front travels slowly into the denser oven-dried matrix leading to a controlled release of the drug [81].

## 3. Future Perspectives: What Is Next?

Native and functionalized BC is considered a high appealing biopolymer by the global market and there is an increasing demand for green biomaterials from bacterial sources rather than extracting these from plant-based sources. BC is a highly pure and biocompatible material thus capable of evading adverse tissue reactions, promoting cellular interaction and tissue development and it can be modified for a variety of applications including wound dressing, modulating drug delivery, scaffolds, medical devices. Many recent studies proved the potential applicability of BC in these biomedical areas using the unique properties of BC (Table 2). Over the last decade, recent advances in genetic and metabolic engineering, supported by model-driven and statistical approaches, allowed a considerable progress on systematic understanding of BC production platforms. However, industrial use of BC still faces obstacles primarily due to the high cost of scaling up production.

Genome-based modeling approaches are crucial for determining the potential gene targets that have effect on BC biosynthesis pathway. Yet, applicability of kinetic-based genome-scale models on bioprocessing needs to be extended, to overcome limitations in systems biology applicability to bioprocess scaling-up. Integrating regulatory networks into models related to bioprocessing may increase the predictive capability of systems biology. However, reliable global gene regulatory networks modeling remains a prohibitively complex computational problem.

Engineering BC producer strains have shown the possibility of not only improving the BC yields but also altering the properties of BC and production of co-polymers. With the aid of recently developed genetic toolkits, the efforts of the scientists are focusing on adding new functional properties to BC. One of the most important aspects seems to be engineering protein secretion mechanism in cellulose-producing strains via synthetic biology. This approach can make it possible to produce BC attached with high value enzymes or proteins targeting specific molecules and leads to additional properties that can go far beyond the properties of original BC. Currently, bacterial cellulose as a regenerative medicine and drug delivery material faces difficulties in modulating drug release and loading patterns. Moreover, mammalian cells are not able to attach to the cellulosic surfaces due to their hydrophilic nature and low non-specific protein adsorption. Cell adhesion to cellulosic materials can be improved by the addition of matrix ligands and surface modifications. It is possible to tune porosity, thickness, and interconnectivity of BC materials, however, toxicity of these chemically treated materials is a concern for biomedical applications. Low immunogenicity and high biocompatibility of BC is retained when using recombinant proteins to tune the properties of BC instead of chemical treatments. Therefore, strain engineering approaches represents enormous potential for promoting the use of BC materials in biomedical applications. Another important aspect that needs to be addressed is the sterilization of BC materials attached with enzymes or proteins. Approaches such as autoclaving or radiation may result in denaturation of these functional proteins.

Current biomedical use of BC is based on manufacturing commercially at the low-medium scale level in moist form which is easily available for use. More efforts still need to be also focused on commercial production of bacterial cellulose at the industrial scale. Bioprocessing of BC production in large scale should be supported by statistical tools to define optimum bioprocess conditions for newly identified media components, BC producing strains, or designs of bioreactors. Use of waste materials from agricultural activities is an area that needs to be explored further. The potential of BC produced by utilization of low cost feed-stocks is considered beneficial in terms of economics, environment, and practicality.

The importance of interdisciplinary research in the area of BC biosynthesis platforms is being noticed among scientists in the last a few years. With collaborative efforts of chemical engineers, biologists, and materials scientists, BC will continue to be a biomaterial of preference, leading to cost-effective production of tailor-made BC materials for biomedical applications soon. Various applications would motivate more and more people to set up factories producing native bacterial cellulose as well as cellulose-based composites. This will also lessen the requirement of plant-derived cellulose proving to be an eco-friendly approach.

## Figures and Tables

**Table 1:** Different approaches used for engineering BC biosynthesis platforms.

Strain	Aim	Approach	Outcome	Reference
*K. sucrofermentans* BPR 2001	sucrose synthase expression to enable sucrose metabolism	overexpression by pSA-SD derived from pSA19	2 times increased BC yield (8 g/L)	[28]
*Acetobacter* ITDI 2.1	β-galactosidase expression to enable lactose metabolism	lacZ genome integration	28-fold increased BC yield ability to use lactose and whey as carbon source	[31]
*A. xylinum* BCRC12334	*Vitreoscilla* hemoglobin (VHb) expression to promote O_2_ utilisation	constitutive expression of VHb by pBla-VHb-122 derived from pBBR122	2-fold increased BC production 50% increased growth rate	[32]
*A. xylinum* BCRC12334	d-Amino acid oxidase (DAAO) expression and immobilization	inducible expression of DAAO by pLacDAAO-122	self-immobilization of DAAO+ cells (10% activity)	[33]
*G. xylinus* 10245	to incorporate N-acetylglucosamine (GlcNAc) sugar residues into glucan chains	overexpression of AGM1, NAG5 and UAP1 via pBBR-GlcNAc	cellulose-chitin copolymer synthesis	[34]
*E.coli* XL1-Blue	BC biosynthesis in *E. coli*	heterogeneous co-expression of BcsA, BcsB and DGC (diguanyl cyclase)	reconstitution of cellulose synthase no BC crystallization	[36]
*E.coli* C41 (DE3) *E.coli* HMS174(DE3)	BC biosynthesis in *E. coli*	heterogeneous expression of bcsABCD operon and upstream operon (*cmcax*, *ccpAx*) via inducible pCMP and pBCS	large fibres with diameters ranging from 10 to 20 μm rapid BC production and short culturing period	[37]
*K. rhaeticus* iGEM	building genetic toolkit for *Acetobacteraceae*	identification of plasmid backbones, characterisation, and engineering of constitutive and inducible promoters	toolkit achieved biosynthesis of patterned cellulose, functionalization of the cellulose surface with proteins, and tunable control over cellulose production	[38]
*G. xylinus* ATCC 700178 *G. hansenii* ATCC 53582 *K. rhaeticus* iGEM	building an expanded genetic toolkit for *Acetobacteraceae*	characterisation multiple natural and synthetic promoters, ribosome binding sites, terminators, and degradation tags by expressing RFP1 reporter gene CRISPRi targeting endogenous acs operon (*acsAB* and *acsD*)	expanded toolkit readily mix-and match for expression modified cellulose with variable chitin content via high or low expression plasmids	[39]
*K. xylinus* CMCC 2955	structural characterisation of BC under various *galU* expression	CRISPRi to downregulate *galU*	porosity increased by 0.5-fold with *galU* repression crystallinity increased with the rise in *galU* expression	[40]
*G. xylinus* BCRC12334	reducing gluconic acid production by eliminating the membrane-bound glucose dehydrogenase (GDH) activity	GDH knock-out	40 and 230% increased BC production in static and shaken culture	[41]
*K. xylinus* DSM 2325	identifying the effect of *pfk*A gene in glucose metabolism	*pfk*A genome integration CRP overexpression by pIN01-crp	increased cellulose production (from 3.5 to 4.5 g/L) sharp decrease in the yield of gluconic acid (from 64.8 to 39.2%)	[42]
*K. xylinus* CGMCC 2955	exploring the effect of oxygen tension on BC production	constitutive expression of VHb by pBla-VHb-122 derived from pBBR122	increased BC yield 26.5 and 58.6% at oxygen tensions of 10 and 15%	[43]
*Enterobacter* sp. FY-07	production of colanic acid to improve water holding capacity of BC	overexpression of wca operon (encoding colanic acid) via inducible pTSK1-tac	water holding capacity enhanced slightly by 1.7-fold	[44]
*K. rhaeticus* and *S. cerevisiae* *co-cultures*	developing ELM system programmed for dedicated tasks	co-culturing of *K. rhaeticus* with engineered strains of *S. cerevisiae* to secrete enzymes into BC or creating living materials that can sense and respond to environmental stimuli	enzyme-functionalized BC, altered physical properties and produced BC-based ELMs that can sense and respond to chemical and optical stimuli	[45]
*K. hansenii* ATCC 23769	investigating the effect of motility genes (*motA* and *motB*) on BC structure	overexpression of MotA and MotB disruption of *motA* and *motB*	substantial loosening of intra-membrane structure overexpression of motility proteins, compact BC structure achieved via disruption of these genes	[46,47]

**Table 2:** Recent studies investigating the potential of BC in biomedical applications.

Application Area	Properties of BC	Reference
Wound dressing	non-toxic, non-carcinogenic and biocompatible, capacity to retain moisture, allows for oxygen exchange	[82,83,84,85]
Drug delivery	nanofibrillar structure represent a suitable macromolecular support for inclusion of drugs and therefore modulation of the drug release	[86,87,88,89,90]
Tissue regeneration/ scaffolds	allows cellular adhesion and proliferation, customizable to control its features	[91,92,93,94]
Vascular grafts	represents high mechanical strength and microporosity	[95,96]
